# DyEndoVO: scene dynamics-aware pose estimation of endoscope in minimally invasive surgery

**DOI:** 10.1007/s11548-025-03549-0

**Published:** 2025-12-22

**Authors:** Jinjing Xu, Reuben Docea, Micha Pfeiffer, Martin Wagner, Marius Distler, Stefanie Speidel

**Affiliations:** 1 National Center for Tumor Diseases (NCT/UCC), Dresden, Germany; 2 The Centre for Tactile Internet with Human-in-the-Loop (CeTI), TU Dresden, Dresden, Germany; 3 University Hospital Carl Gustav Carus, Dresden, Germany; 4 German Cancer Research Center (DKFZ), Heidelberg, Germany

**Keywords:** Endoscope localization, Visual odometry, Motion detection, Tissue deformation

## Abstract

****Purpose**:**

Estimating the 6 degrees of freedom (DoF) pose of an endoscope is crucial for various applications in minimally invasive computer-assisted surgery. Image-based approaches are some of the most practical solutions for pose estimation in surgical environments, due to a limited workspace and sensor constraints. However, these methods often struggle or fail in dynamic scenes, such as those involving tissue deformation, surgical tool movement, and tool-tissue interaction.

****Methods**:**

We propose DyEndoVO, an end-to-end visual odometry method in dynamic endoscopic scenes. Our method consists of a transformer-based motion detection network and a weighted pose-optimization module. The motion detection network infers scene dynamics and guides the pose estimation. Furthermore, we introduce a semi-synthetic dataset featuring tissue and tool movement categories. It serves as training data, improving pose estimation accuracy, and also includes motion masks to enable a fine-grained inspection and evaluation.

****Results**:**

DyEndoVO significantly outperforms state-of-the-art methods in pose estimation for dynamic surgical scenes. Despite being trained solely on a synthetic dataset, our method generalizes well to real-world data without fine-tuning. Further analysis attributes this success to the effective detection of scene dynamics and the adaptation in the learned weight toward pose estimation; moreover, the semi-synthetic dataset also plays a key role in bridging the sim-to-real gap.

****Conclusions**:**

In this work, we aim to improve the accuracy and robustness of pose estimation in challenging dynamic surgical scenes, by effectively handling scene dynamics. Our method, combined with the proposed synthetic dataset, demonstrates improved performance in pose estimation and generalizes well to real-world data, showing its potential in advancing related works such as SLAM and 3D reconstruction in complex surgical environments.

**Supplementary Information:**

The online version contains supplementary material available at 10.1007/s11548-025-03549-0.

## Introduction

Visual odometry (VO), as an essential component of pose estimation in visual Simultaneous Localization and Mapping (vSLAM) systems, has been widely researched in rigid scenes [1, 2]. It can be deployed in minimally invasive surgery (MIS) to facilitate endoscope navigation, 3D reconstruction of the abdominal scenes and augmented reality (AR) visualization of preoperative data. However, owing particularly to ambiguities arising from the complex behavior and interaction between non-rigid tissues and surgical tools, the problem of endoscope pose estimation in dynamic MIS scenes remains unsolved. In this work, we tackle this problem of Visual Odometry in MIS. We do so through introducing a new VO method, as well as a new dataset to supplement training and evaluation. Our main contributions are as follows:

- We propose DyEndoVO, an end-to-end VO method that integrates a transformer-based motion detection network with a weighted pose-optimization module. We build upon the state-of-the-art motion detection network, OCLR [3], and recent work from Hayoz et al. [4] in differentiable pose optimization.
- We construct a semi-synthetic stereo dataset, DynaSCARED, which enhances the training of our method. The dataset also enables a fine-grained evaluation of model performance. This is owed to its categorical scenario sequences with ground truth scene motions masks, which are typically unavailable for real data.
- We thoroughly evaluate our method on two datasets: our own, DynaSCARED, and an in vivo surgical dataset StereoMIS [4]. Despite being trained solely on DynaSCARED, our method generalizes well, shows by its outperforming of baselines in most cases. We verify that the success of our method stems from its ability to effectively detect scene dynamics and adapt the learned probability map, aiding in accurate pose estimation in dynamic environments.

## Related work

Pose estimation methods can be categorized as learning based and structure based. Among learned methods, PoseNet [5] directly regresses the absolute pose and therefore fails to generalize across scenes. Relative pose regression methods [6, 7] are proposed to overcome this limitation, but are still limited in accuracy compared to conventional pose-optimization methods, in which either sparse keypoint matches [1] or dense data-associations [2, 8] are explicitly established. However, conventional optimization methods are non-differentiable, and therefore of limited use in end-to-end learning. Recent works [4, 9, 10] demonstrate the potential of deep declarative networks (DDN) [11] to make the problem of pose optimization differentiable. This enabled a new class of hybrid end-to-end VO methods that, unlike direct pose regression, use differentiable optimization to train deep networks for geometric tasks. Notable examples include DSAC [12], which introduced a differentiable RANSAC for robust localization, and complete VO/SLAM systems like DROID-SLAM [13] and DPVO [14], which leverage differentiable bundle-adjustment-like backends for end-to-end refinement of pose and geometry.

Despite containing approaches to handling outliers, state-of-the-art VO and SLAM methods [1, 2] often struggle in dynamic environments, as they make assumption of scene-rigidity. The broader computer vision and robotics community handles dynamics by excluding elements. One strategy, object detection [15, 16], masks semantic classes (e.g., people). To avoid this method’s false exclusions, moving consistency checks use geometric constraints based on optical flow [17, 18] to reject features inconsistent with ego-motion. Surgical scenes are among the most challenging environments due to their non-rigid and dynamic nature, where tissue deforms and surgical tools move. Among attempted methods for localization in MIS, ArthroSLAM [19] works inside the relatively rigid human knee joint for orthopedic surgery. Other SLAM methods [20–22] assume negligible tissue deformation in the MIS scene. There also exist works [23–27] which handle tissue deformation by modeling the tissue surface; of which most recent progress [28, 29] leverages Gaussian Splatting [30] in MIS SLAM system. Another approach is to mask out dynamic objects - such as moving cars - automatically and use only the remaining static scene for pose optimization, as done in Dynaslam [16]. Similarly, Hayoz et al. [4] ignore correspondences on surgical tools with a pretrained tool segmentation model. However, such a semantic-priors-based approach may discard valuable information, e.g., a temporarily still surgical tool will be masked out, despite being reliable for anchoring pose estimation. Moreover, Hayoz’ pretrained model accounting only for surgical tools. Motivated by the above, we explore the potential of motion detection for accurate localization in dynamic scenes.

## Method

### Overview

We tackle the problem of stereo visual odometry, which takes consecutive pairs of stereo images $\{\textbf{I}^{L}_{t}$, $\textbf{I}^{R}_{t}\}$ and $\{\textbf{I}^{L}_{t+1}$, $\textbf{I}^{R}_{t+1}\}$, and estimates the relative pose $\textbf{P}^{*}_{t+1,t}$ between the consecutive left images, $\{\textbf{I}^{L}_{t+1}$, $\textbf{I}^{L}_{t}\}$. We chain relative poses to obtain full trajectories as in [4].

**Table 1:** Experimental results on DynaSCARED test split. We categorize 8 scenarios based on camera status and scene dynamics: “S0” / “S1” indicate moving / still camera; “w.Tool” / “w.Tissue” indicate the scene contains tool movement/tissue deformation. The best performances are highlighted in bold. “Naive” refers to non-weighted pose optimization; “motion” indicates the motion model as defined in Sec. 3.2.3; “rf.W” stands for further refinement of the optimized pose with weighted inliers (we detail it in the discussion); and “rf.N” stands for vanilla refinement. “res.3D” represents optimization is based on 3D residual as referred in Sec. 3.2.2. Notice the IoU for “Static Scene” is not reported due to its static nature

		w.Tool & w.Tissue				w.Tool				w.Tissue				Static Scene		
		RPE	RPE	ATE	IoU	RPE	RPE	ATE	IoU	RPE	RPE	ATE	IoU	RPE	RPE	ATE
		$\left[ mm\right] \downarrow$	$\left[ {deg }^{}\right] \downarrow$	$\left[ mm\right] \downarrow$	$\left[ \% \right] \uparrow$	$\left[ mm\right] \downarrow$	$\left[ {deg }^{}\right] \downarrow$	$\left[ mm\right] \downarrow$	$\left[ \% \right] \uparrow$	$\left[ mm\right] \downarrow$	$\left[ {deg }^{}\right] \downarrow$	$\left[ mm\right] \downarrow$	$\left[ \% \right] \uparrow$	$\left[ m\right] \downarrow$	$\left[ {deg }^{}\right] \downarrow$	$\left[ mm\right] \downarrow$
S0	Naive	0.559	0.489	16.619	–	1.120	0.794	28.670	–	0.332	0.322	27.780	–	0.111	0.107	5.573
	Ours (motion)	0.177	0.161	**7**.**645**	96.4	0.568	0.394	15.551	70.9	0.125	**0**.**111**	**5**.**552**	87.2	**0**.**111**	**0**.**107**	5.590
	**Ours**	**0**.**147**	**0**.**140**	7.695	84.8	**0**.**354**	**0**.**251**	**11**.**371**	53.4	0.125	0.113	5.632	78.4	0.112	0.108	**5**.**545**
	Ours(rf.N)	0.375	0.357	7.990	–	0.850	0.600	19.401	–	0.180	0.166	9.684	–	0.105	0.102	4.340
	**Ours(rf.W)**	**0**.**135**	**0**.**132**	**4**.**860**	–	**0**.**321**	**0**.**228**	**8**.**578**	-	**0**.**112**	**0**.**105**	**3**.**798**	-	**0**.**105**	**0**.**102**	**4**.**326**
	Ours(res.3D)	0.342	0.310	20.920	–	0.516	0.374	13.657	–	0.615	0.492	19.187	–	0.396	0.314	8.759
S1	Naive	0.332	0.392	13.452	–	0.736	0.628	16.193	–	0.064	0.041	2.568	–	0.001	0.000	0.157
	Ours (motion)	0.103	0.095	2.932	95.8	0.306	0.244	9.914	72.6	0.003	0.000	0.212	94.4	0.001	0.000	0.154
	**Ours**	**0**.**068**	**0**.**058**	**2**.**363**	84.8	**0**.**138**	**0**.**106**	**6**.**900**	55.4	**0**.**003**	0.001	**0**.**175**	80.0	0.001	0.000	0.163
	Ours(rf.N)	0.112	0.130	4.404	–	0.277	0.216	9.707	–	**0**.**002**	**0**.**000**	**0**.**174**	-	**0**.**001**	**0**.**000**	**0**.**163**
	**Ours(rf.W)**	**0**.**065**	**0**.**055**	**1**.**952**	–	**0**.**103**	**0**.**078**	**3**.**950**	-	**0**.**002**	**0**.**000**	**0**.**174**	-	**0**.**001**	**0**.**000**	**0**.**163**
	Ours(res.3D)	0.097	0.071	3.293	–	0.057	0.031	3.175	–	0.110	0.067	1.711	–	0.011	0.000	0.816

Our proposed network contains two sub-modules: a transformer-based motion detection module build upon [3], followed by a geometry-based differentiable pose-optimization module inspired by [4]. The latter performs weighted pose estimation with a motion signal from the motion detection module and leverages DDN for end-to-end training. We follow the same practice as in [4] of using RAFT [31] to obtain temporal data-association and stereo depth for pose estimation. To train our model, we introduce the semi-synthetic dataset DynaSCARED, similar to [3], but with significant improvements in the coupling of stereo images and pose labels, along with surgery-specific details (outlined in Sec. 3.3). Our framework is motivated by the objective of learning motion representations exclusively from optical flow inputs under direct pose supervision, aiming to improve estimation accuracy in dynamic minimally invasive surgery (MIS) environments. This goal informs our adoption of OCLR, a SoTA motion detection network, as the foundation of our motion detection module, and motivates our incorporation of a trainable optimization module facilitating direct learning in end-to-end manner. In the following sections, we present the details of our method in Sec. 3.2 and the key components of our synthetic data generation pipeline in Sec. 3.3.

### Motion-aware pose estimation

#### Motion detection module

Our motion detection module is built upon [3], consisting of CNN-based U-Net architecture with a transformer-based bottleneck. We refer to [3] for the original design, and only clarify major adaptions regarding the target task and the architecture.

**Different target tasks**. OCLR [3] is proposed for object-wise amodal segmentation, with a sequential input of *N* frames. Additionally, they predict ordering in layers for overlayed objects. On the other hand, our module, given an input of consecutive frames (N=2), estimates the relative pose for the input image pair. To this end, we reformulate the task for our motion module to be the inference of all present motion signals as a whole, while omitting concerns in addressing object occlusions and layer ordering. Furthermore, we adapt the learned probability to serve specifically weighted pose estimation in dynamic scenes. We achieve this by architecturally changing the motion module as stated below, and by using direct supervision with pose loss as detailed in Sec. 3.2.3.

**Task-specific architecture adaptation**. To suit our target task, we remove the MLP-based layer predictor, as it is irrelevant for our target task. Moreover, we abandon their design idea in establishing one-to-one correspondence for objects and the learned queries, proposing instead to learn all motion signals with one single motion query. The adaptation is also intuitively critical in the surgical context: the detection of deforming and manipulated tissue entails ambiguous and uncountable “objects” for one-to-one mappings, thereby potentially making their method fail in detecting tissue deformation as shown in rows *A* and *C* of Fig. 4. Our formulation naturally overcomes the limits in numbers of moved objects to be detected.

#### Differentiable pose estimation

To enable end-to-end training of our motion module with supervision from poses, we follow the approach in recent work [4], leveraging DDN to make the weighted pose-optimization problem differentiable, as formulated as follows:

1 $$\begin{aligned} \begin{aligned} \textbf{P}_{{t+1},t}^{\star }=\underset{\textbf{P}_{{t+1},t}}{\operatorname {argmin}}\left\{ \sum _{\textbf{x} \in \boldsymbol{\Omega }} (1-\textbf{M}(\textbf{x}))\cdot \textbf{r}\left( \textbf{P}_{{t+1},t}, \textbf{x}\right) ^2\right\} \end{aligned} \end{aligned}$$

where $\textbf{M}$ represents the inferred motion probability from motion module, $\boldsymbol{\Omega }$ represents the set of 2D pixel coordinates $\textbf{x}$ in image $\textbf{I}^{L}_{t}$, optimization target $\textbf{P}_{{t+1},t}$ denotes relative pose between frames. We adopt the widely used reprojection error [1, 2] to formulate the per-pixel optimization residual ${\textbf {r}}$. Given pixel $\textbf{x}$, the residual is computed as:

2 $$\begin{aligned}  &   \textbf{r}\left( \textbf{P}_{{t+1},t}, \textbf{x}\right) =\sqrt{\frac{1}{HW}} \Vert \mathbf {\pi }\left( \textbf{T}_{{t+1},t}\mathbf {\pi ^{-1}}\left( \textbf{D}_t(\textbf{x}), \textbf{x}\right) \right) \nonumber \\  &   \quad -\left( \textbf{x}+\textbf{F}^{fwd}_t(\textbf{x})\right) \Vert _2 \end{aligned}$$

where $\textbf{D}_t$ is the depth map of the current frame $\textbf{I}^{L}_{t}$, $\mathbf {\pi }$ represents camera projection process from 3D pointcloud to image plane and $\mathbf {\pi ^{-1}}$ denotes the inverse projection. The transformation matrix $\textbf{T}_{{t+1},t}$ projects 3D points from frame $\textbf{I}^{L}_{t}$ to frame $\textbf{I}^{L}_{t+1}$. We follow the optimizer choice and hyper-parameters in [4]. It is worth noting that we adopt different residuals $\textbf{r}$ compared to Hayoz et al. [4]. Specifically, our method does not contain the noise-sensitive 3D alignment error but only a 2D reprojection error; consequently our method does not contain a further learned weighting parameter over the two. Our preliminary experiments show that the alignment error does not necessarily contribute to weight map learning in our method, as we report in Table 1.

#### Loss design and supervision

Our network is trained end-to-end with supervision from ground truth relative poses. The loss function is formulated as follows:

3 $$\begin{aligned} \textbf{L}_{\text{ pose }}=\left\| \textbf{P}_t^{\star }-\textbf{P}_t^{\textbf{gt}}\right\| _1 \end{aligned}$$

For ablation purposes, we train motion models with ground truth motion masks if available, and formulate the loss as follows:

4 $$\begin{aligned} \textbf{L}_{\text{ motion }}=BinaryCrossEntropy(\textbf{M}_t^{\star },\textbf{M}_t^{\textbf{gt}}) \end{aligned}$$

Unless otherwise indicated, we refer the end-to-end pose models trained with $\textbf{L}_{\text{ pose }}$ as our *model*; and models trained with $\textbf{L}_{\text{ motion }}$ as *motion* *models*.

### Synthetic data generation

**Fig. 1:**
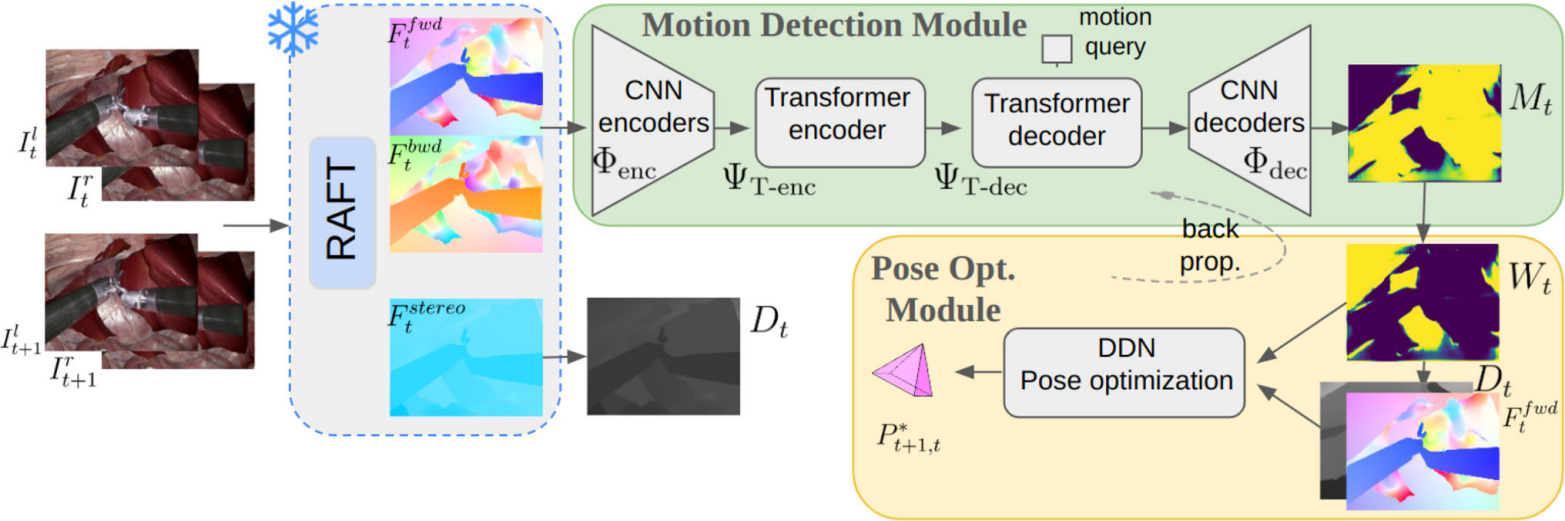
**DyEndoVO Overview.** Firstly, given $\{\textbf{I}^{L}_{t}$,$\textbf{I}^{L}_{t+1}\}$, the pretrained RAFT network computes forward and backward temporal optical flows, $\textbf{F}^{fwd}_{t}$ and $\textbf{F}^{bwd}_{t}$, as well as stereo flow $\textbf{F}^{stereo}_{t}$ from $\{\textbf{I}^{L}_{t}$, $\textbf{I}^{R}_{t}\}$. From $\textbf{F}^{stereo}_{t}$ we obtain depth $\textbf{D}_{t}$, and from $\textbf{F}^{fwd}_{t}$ and $\textbf{F}^{bwd}_{t}$ we obtain data-association in our VO method. Our motion detection module takes $\{\textbf{F}^{fwd}_{t}, \textbf{F}^{bwd}_{t}\}$ as input and predicts a pixel-wise scene dynamics (motion) probability $\textbf{M}_{t}$, from which a complementary weight map $\textbf{W}_{t}$ is then formed. $\textbf{W}_{t}$ then serves as guidance for weighted pose optimization to estimate the relative pose, $\mathbf {{P}^{*}_{{t+1},t}}$, as detailed in Sec. 3.2.2. The network is trained in an end-to-end manner with direct supervision from ground truth relative poses

**Fig. 2:**
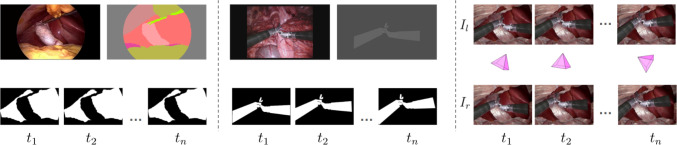
**Example on motion simulation and composed sequence. ** From left to right: ground truth motion masks for simulated non-rigid deformation of sampled *liver* patch; motion masks for simulated rigid tool movement; composed stereo sequences (denoted as $I_{l}$, $I_{r}$) coupled with ground truth camera trajectory

To facilitate end-to-end training of our model, we propose a semi-synthetic dataset DynaSCARED, comprising short (stereo) sequences with scene motion masks, camera trajectory and camera parameters. The dataset generation pipeline builds upon a foreground-background layer composition scheme proposed in [3], while tailored for our task in the following ways: 1) **Scene-related motion simulation and surgical specification**. Our data simulates rigid and non-rigid motion depending on the foreground content (surgical tools and soft tissues). 2) **Semi-realism**. The foreground patches are realistic in both shape and appearance (we extract them randomly from Robotic Instrument Segmentation [32] for tool patch; from CholecSeg8k [33] for tissue patch), while the background also represents the actual movement of handheld endoscope (we obtain the background from SCARED [34]). 3) **Fine-grained categorization**. We categorize our data to 8 scenarios based on motion status of the camera and foreground surgical tools and soft tissue, which enables us to train with diverse and well-distributed data and allows for fine-grained evaluation. We adopt homography transformation on tool patch for movement simulation of surgical tools, and leverage thin plate splines on tissue patch for deformation simulation of non-rigid soft tissue. We refer Sec. A.2, A.3 and A.4 in supplements for data generation details of crucial components. We include an example of a generated moving camera sequence containing simulated tissue deformation and tool motion as in Fig. 2. We highly recommend the link in Sec. A.1 of supplements for viewing our data samples.

## Experiments

### Datasets and metrics

To benchmark visual odometry and motion detection, we use the DynaSCARED test split, which includes 100 sequences (300 frames each) across 8 scenarios. To demonstrate our model’s generalization, we perform extensive experiments on StereoMIS [4], a robotic-assisted MIS dataset with realistic scene dynamics and labeled endoscope poses. We also assess DynaSCARED’s effectiveness in training our model by comparing performance when trained on: 1) a synthetic motion detection dataset from [3] (SynMotion), and 2) the StereoMIS training split from [4]. We report relative position error (RPE) and absolute trajectory error (ATE) to evaluate pose estimation accuracy. Additionally, we report Intersection over union (IoU) on the inferred motion probability *M* (with ground truth motion in DynaSCARED) to quantitatively assess motion detection, aiding in pose estimation diagnostics. For qualitative analysis, we visualize the learned weight map *W*, which complements *M*.

### Experimental setup

We train our model on DynaSCARED (training & validation contains 4000/500 sequences with 30 frames each) with a batch size of 8. We use the Adam optimizer with a learning rate of $1 \times 10^{-5}$. with a warm up-decay strategy as in [3]. All our ablated models are trained on a single 32GB NVIDIA RTX V100 GPU with 7000k iterations. We augment the dataset with color jittering and blurring, coupled with the frame skipping strategy of [3] during training. For the differentiable pose optimizer, we set 30 iterations as the optimization stopping limitation across all experiments.

### Experimental results

#### Evaluation on DynaSCARED and ablations

In this section, we evaluate our method on the test split of DynaSCARED. In addition, we ablate critical modifications in our method, and conduct an experiment to justify the importance of adopting end-to-end training. In addition to RPE and ATE, we analyze the improved pose estimations performance with the assisted motion detection metric IoU. The scenes are categorized based on the motion status of the scene and camera.

**Table 2:** Experimental results on StereoMIS snippets containing camera motion. N denotes the number of input frames ($N=2$ unless stated, i.e., consecutive pair of input frames). The best are highlighted in bold

		Bowel: 3796 frames			Liver: 5174 frames		
		RPE	RPE	ATE	RPE	RPE	ATE
		[mm]$\downarrow$	[deg]$\downarrow$	[mm]$\downarrow$	[mm]$\downarrow$	[deg]$\downarrow$	[mm]$\downarrow$
Visual Odometry	Hayoz et al. [4]	0.164	0.116	1.581	0.409	0.237	3.937
	EDaM [35]	**0**.**152**	**0**.**104**	1.405	0.406	0.234	3.748
	OCLR [3]	0.154	0.107	1.461	0.348	0.190	3.321
	Ours	0.153	0.106	1.452	**0.344**	0.189	**3**.**268**
	Ours(rf.W)	**0**.**152**	**0**.**104**	1.444	**0**.**344**	**0**.**186**	4.191
	Ours (motion)	0.155	0.107	1.490	0.346	0.190	3.287
SLAM	ORBSLAM3 [1]	0.222	0.145	**1**.**102**	0.469	0.288	3.676
	ElasticFusion [2]	0.253	0.162	1.577	0.415	0.246	3.499
	DroidSLAM [13]	0.205	0.144	1.185	0.807	0.297	6.585

We summarize our ablations as follows: 1) Compared to *Naive*, where the inferenced weight map in *Ours* is replaced with a constant weight map of 1, *Ours*(end-to-end pose model) outperforms across all dynamic scenarios, both with a fixed camera (*S*1) and a challenging moving camera (*S*0). This is due to effective detection of scene dynamics, including rigid tool movement and non-rigid tissue deformation, as reflected by high IoU values. 2) We show that using the 3D alignment (Ours(res.3D)) error instead of a 2D reprojection error (Ours) significantly degrades performance, which justifies our design for target residual as referred in Sec. 3.2.2. 3) Comparing *Ours*(*rf*.*W*) to *Ours*, we see improved performance with our proposed test time refinement strategy as detailed in Sec. 3.2.2, while the vanilla refinement *Ours*(*rf*.*N*) shows worse performance compared to *Ours*, reinforcing the value of our learned weights.

#### Evaluation on in vivo dataset

In this section, we compare our method with five baseline methods and one OCLR−extended [3] method, including rigid SLAM methods ORBSLAM3 [1] and ElasticFusion [2] following the choices in recent work [4]; and non-rigid methods Hayoz [4] and additional one extended upon OCLR [3], which are most close to ours in methodology. We further compared our method with endoscope tracking method EDaM [35] and a learning based SLAM method DroidSLAM [13]. To be specific, we extend the motion detection method OCLR for pose estimation purposes, by replacing the adopted learned weight map in weighted pose optimization, with inferred motion probability.

Notice the endoscope in StereoMIS is often still, to sufficiently benchmark methods, from which we extract short snippets (26 frames each), captured when camera is moving; we group the obtained snippets to liver/bowel scene (5174/3796 frames) and report the evaluation results in Table 2. For completeness, we also experiment on still-camera snippets as reported in Table 3, where the snippets are grouped into scenario without presence of surgical tools (containing breathing and pulsation) and scenario involving severe tool-tissue interaction. The former scenario is further grouped to liver/bowel scene(2600/1326 frames), while we observe the second only captures bowel scene therefore we grouped based on their source sequence P2_6/P2_7 1066/1248 frames). we refer Sec. C.1 in supplements for detailed strategy in obtaining the snippets.

**Fig. 3:**
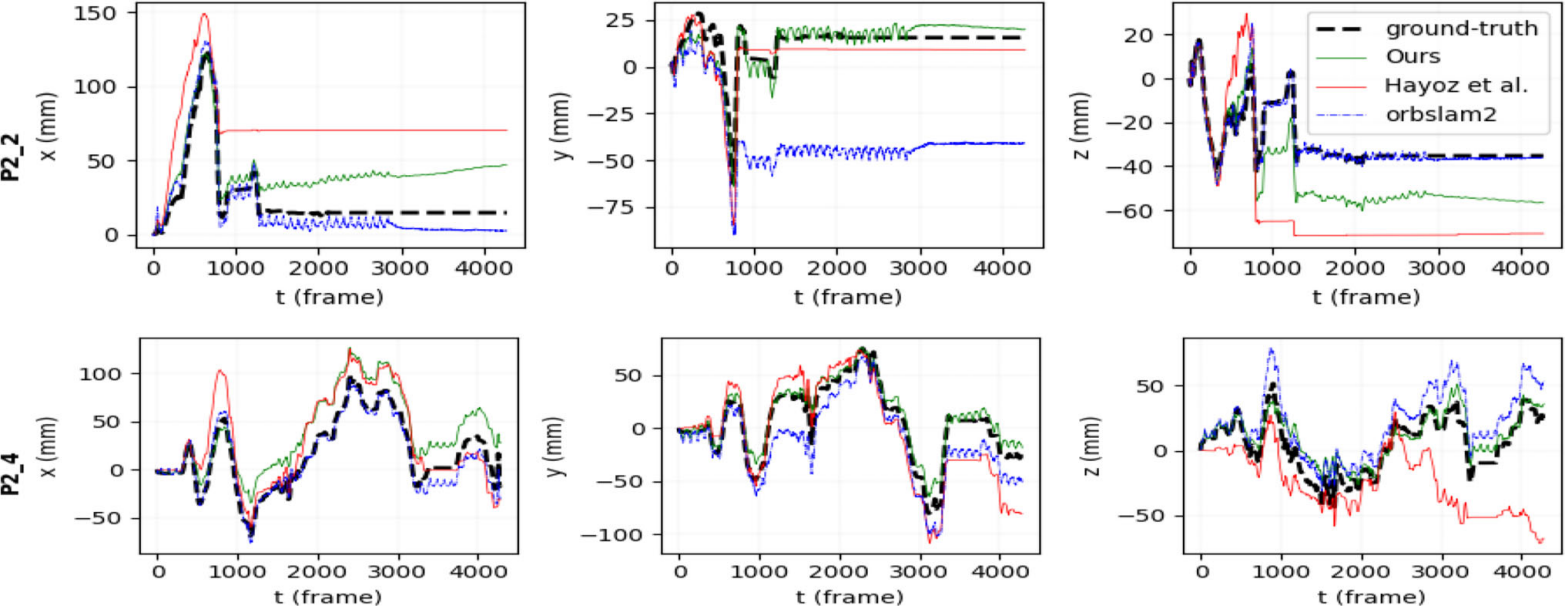
**Trajectories on full StereoMIS sequences.** In sequence P2_2 (up), there are extreme scene dynamics (endoscope out of trocar in frame 805, quickly reentering), leading briefly to no anchoring for relative pose estimation, and consequently tracking failure until end. Our method shows strong relative pose estimation, indicated by 1) a similar trajectory shape to that of ground truth 2) the reported mean RPE metrics. In sequence P2_4 (down), our method is the only one which consistently tracks

**Fig. 4:**
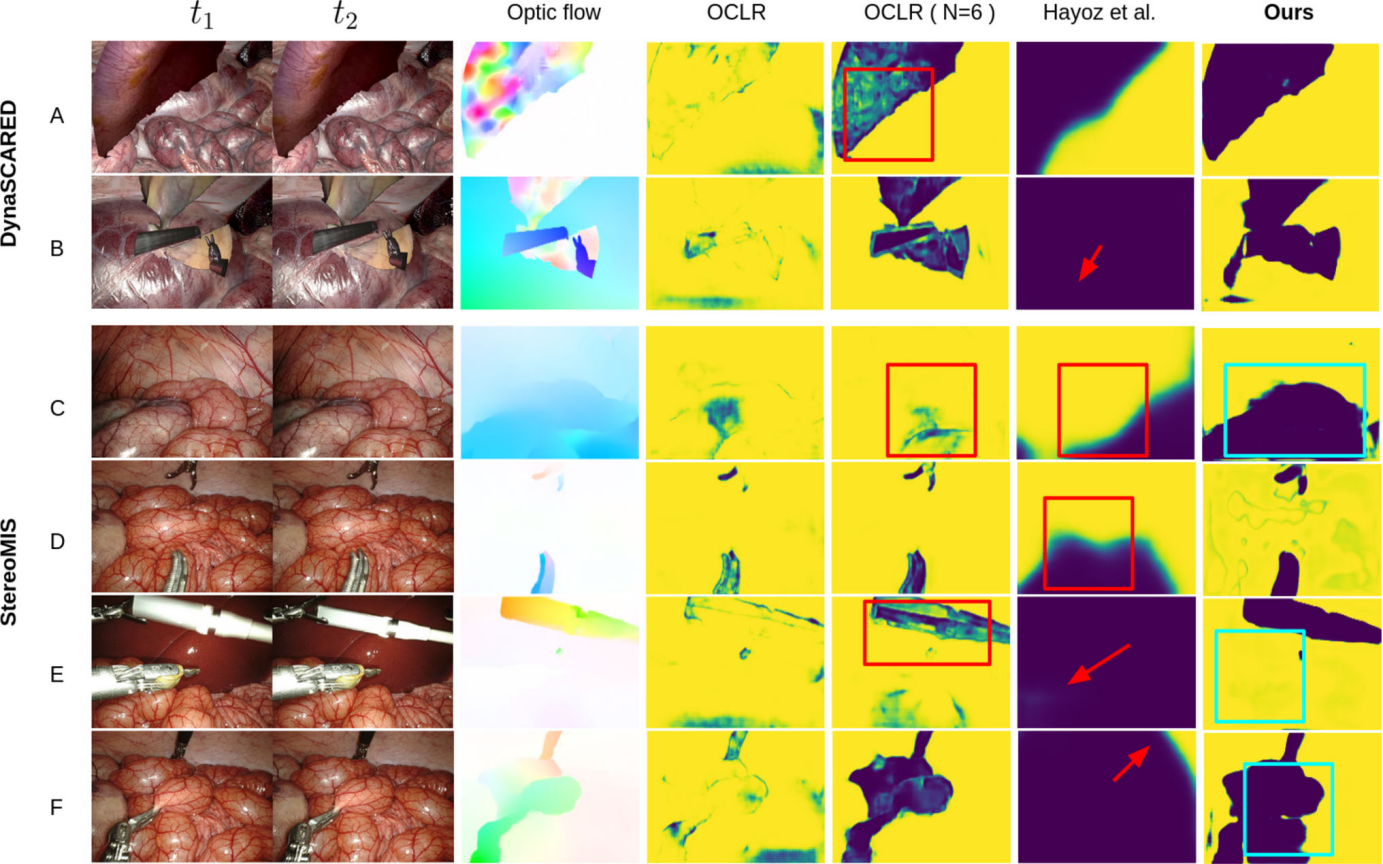
**Visualization of learned weight map.** Row *A* & *B* represent example results on DynaSCARED. Other results are on StereoMIS. Values in the weight maps are on a color scale from blue (small) to yellow (large). We mark regions containing non-ideal/reasonable inference with red/cyan boxes. Red arrows indicate over-sensitivity to motion signals. We refer Fig. 1 and the video link in the supplementary for more examples and enhanced visualization

For evaluations conducted on snippets captured with moving camera as reported in Table 2. , our model outperforms Hayoz et al. [4] significantly in both RPE and ATE when camera moves as shown in Table 2. This is likely due to the well simulated and distributed motion signals in our training data (DynaSCARED), and our transformer-based architecture, adapted from the state-of-the-art motion detection network OCLR [3], which captures scene ambiguities better than their CNN. As shown in Fig. 4, Hayoz’s model can fail in properly attenuating colon deformation (row *C*) and is overly sensitive to motion signals (large areas around the moving tool are attenuated in rows *D*-*F*), potentially wasting information. Compared with the visual odometry method extended upon OCLR [3], our model shows improved detection of non-rigid tissue deformation (e.g., row *A* *C*, *F* in Fig. 4). This is likely because OCLR’s training data are less surgery-specific and designed for amodal motion segmentation, a binary task, unlike our focus on pose estimation; on top of that, the end-to-end training with pose supervision potentially also contribute toward the target pose estimation task, as demonstrated with the deteriorated performance of Ours (motion) in Table 2, Table 1. Notice our method also outperforms EDaM [35], an visual odometry approach proposed for endoscopic videos, it can handle tissue deformation to an extent, while it assumes slow tissue deformations. We also put our methods in comparison with SLAM methods, our method generally performs better due to its ability to handle scene dynamics, though it, like other VO baselines, underperforms in ATE due to the lack of global optimization. For evaluation conducted on snippets captured with static camera (involving extensive tissue breathing, pulsation, and tool-tissue interactions), as reported in Table 3, our method achieves overall best performance among prior-free baselines, while ranks second to Hayoz et al.  [4]. We emphasize that their method relies on continuous surgical tool masking to enhance performance. This dependency is demonstrated through an ablation study in tool-tissue interaction scenarios: when evaluated without tool mask preprocessing (denoted as metrics within brackets for Hayoz et al). exhibits significant performance degradation. In contrast, our framework maintains robustness without requiring explicit tool segmentation, establishing it as the most effective prior-free solution for dynamic surgical environments. Whereas as we have argued, consistently masking objects out based on semantic priors regardless of their motion status, can lead to potential information wasting for accurate localization and has limitations in generalization across unseen tool categories. On the other hand, as expected, Hayoz et al. [4] shows competitive results on static camera scenario, this is because the method has a tendency of over-sensitivity to motion signals (shown in row F in Fig. 4, more severe in row E), thereafter “prefers” the still camera snippets (the pose optimization is initialized with identity transformation). The results on full sequence align with our conclusion, as shown with the representative trajectory in Fig. 3, and the reported metrics in Table 2 of supplementary material.

**Table 3:** Experimental results on StereoMIS snippets containing no camera motion. The performance of Hayoz et al. without using additional surgical tool masks is denoted within brackets. Other notation follows the same as in manuscript. The best and the second best performances are highlighted as bold and underlined. Notice, DroidSLAM failed for static camera snippts therefore not included in table

Method	W.o surgical tools						Tool-tissue interaction					
	liver: 2600 frames			bowel: 1326 frames			bowel P2_6: 1066 frames			bowel P2_7:1248 frames		
	RPE	RPE	ATE	RPE	RPE	ATE	RPE	RPE	ATE	RPE	RPE	ATE
	$\left[ mm\right] \downarrow$	$\left[ {deg }^{}\right] \downarrow$	$\left[ mm\right] \downarrow$	$\left[ mm\right] \downarrow$	$\left[ {deg}^{}\right] \downarrow$	$\left[ mm\right] \downarrow$	$\left[ mm\right] \downarrow$	$\left[ {deg }^{}\right] \downarrow$	$\left[ mm\right] \downarrow$	$\left[ mm\right] \downarrow$	$\left[ {deg}^{}\right] \downarrow$	$\left[ mm\right] \downarrow$
Hayoz et al. [4]	0.087	**0**.**005**	**0**.**003**	**0**.**006**	**0**.**001**	0.051	**0**.**053**	**0**.**007**	**0**.**366**	**0**.**056**	**0**.**011**	**0**.**615**
		–			–		(0.130)	(0.039)	(1.170)	(0.151)	(0.055)	(1.664)
EDaM[35]	0.097	0.036	0.438	0.054	0.025	0.664	0.404	0.224	4.680	0.363	0.213	4.084
OCLR (N=6)	0.052	0.011	0.299	**0**.**006**	**0**.**001**	0.040	0.213	0.091	2.804	0.215	0.097	2.803
Ours	0.048	0.009	0.270	0.007	**0**.**001**	**0**.**039**	0.112	0.037	1.273	0.111	0.044	1.211
Ours(rf.W)	**0**.**047**	0.009	0.268	0.007	**0**.**001**	**0**.**039**	0.110	0.036	1.213	0.105	0.039	1.118
Ours (motion)	0.053	0.011	0.314	**0**.**006**	**0**.**001**	0.041	0.175	0.069	2.180	0.159	0.067	1.919
ORBSLAM3 [1]	0.152	0.062	**0**.**264**	0.089	0.047	0.208	0.349	0.198	2.986	0.295	0.143	2.532
ElasticFusion [2]	0.304	0.117	1.041	0.158	0.076	1.144	0.436	0.259	5.567	0.368	0.230	4.626
DroidSLAM [13]		–			–			–			–	

#### Analysis the efficacy of DynaSCARED in model learning

Finally, we validate the effectiveness of DynaSCARED by training our model (pose model) and motion model with two other datasets [3, 4] as shown in Table 4. Additionally, to explore the effect of chosen technique for tissue deformation simulation in generation of DynaSCARED, we also compare our choice of thin plate spline technique with alternatives, namely, B-spline based free form deformation and elastic distortion algorithm, and generated two variants of DynaSCARED. We again train our models with the referred variants and report results in Table 4. The evaluation is conducted on StereoMIS snippets containing camera motion.

Compared with models trained with other datasets, as reported in Table 4, our motion model *Ours*(*motion*) outperforms the motion model trained on SynMotion [3], *Ours*(*m*.*SYN*). Our pose model, surprisingly, significantly outperforms a pose model trained on real surgical data StereoMIS *Ours*(*p*.*SM*). The above demonstrates the effectiveness of our exquisitely designed data modeling strategy toward well-distributed, surgery-specific semi-synthetic data as described in Sec. 3.3; and serves as strong evidence for its contribution to the model learning of our method. On the other hand, the outperformance of our pose model over motion model additionally verifies the benefit of leveraging end-to-end learning, despite the practicability of motion supervision with our synthetic DynaSCARED. Compared with models train with other variants (p.DynaSCAREDffd, p.DynaSCAREDed) of DynaSCARED, we observe comparable performance. This is potentially because our motion detection module only takes optic flow as input and the model is directly trained with pose supervision toward motion-aware pose estimation, both reduce the model’s sensitivity to a specific deformation pattern simulated in the training data. Moreover, the target task of pose estimation can also alleviate the tendency of our model in overfitting to the intermediate motion detection, and consequently contributes in bridging the synthetic-real gap.

**Table 4:** Ablate tissue deforming modeling method used in the generation of DynaSCARED. The experiments are conducted on in vivo StereoMIS (moving camera snippets). “m.”/“p.” stands for our pose/motion model, followed with training dataset. (“m.SYN” stands for our motion model trained with SynMotion [3], “p.SM” stands for our pose model trained with StereoMIS [4]). We denote two variants of DynaSCARED adopting B-spline based free form deformation and elastic distortion algorithm for tissue deformation simulation as DynaSCARED-ffd, DynaSCARED-ed

		Bowel: 3796 frames			Liver: 5174 frames		
		RPE	RPE	ATE	RPE	RPE	ATE
		[mm]$\downarrow$	[deg]$\downarrow$	[mm]$\downarrow$	[mm]$\downarrow$	[deg]$\downarrow$	[mm]$\downarrow$
	**Ours (p.)**	** 0.153**	**0**.**106**	**1**.**452**	**0.344**	**0**.**189**	3.268
	Ours (m.)	0.155	0.107	1.490	0.346	0.190	3.287
	Ours (m.SYN)	0.164	0.112	1.598	0.351	0.195	3.374
	Ours (p.SM)	0.179	0.119	1.802	0.356	0.200	3.364
	Ours (p.DynaSCARED-ffd)	**0**.**153**	**0**.**106**	1.457	0.345	**0**.**189**	3.280
	Ours (p.DynaSCARED-ed)	**0**.**153**	**0**.**106**	1.454	**0**.**344**	**0**.**189**	**3**.**260**

## Conclusions

In this work, we make ample progress toward improving the accuracy and robustness of pose estimation in challenging dynamic surgical scenes, by effectively handling scene dynamics. Our method, along with the proposed synthetic dataset, demonstrates improved performance in pose estimation compared to recent state-of-the-art works such a Hayoz et al. [4]. Several additional challenges and potential improvements, beyond the scope of this work, will be addressed in future efforts: 1) Our method obtains data-association from optical flow and depends on its accuracy, which is known to be especially challenging in images with smoke and blur 2) Our method lacks a re-localization module for consistent tracking 3) Our method fails in extreme cases when the scene is completely moved, as it requires the “anchoring” in rigid scene area. In this last case, incorporating an IMU sensor may improve robustness. All in all, our work is a strong contribution to odometry in MIS, and holds promise for also advancing SLAM and 3D reconstruction in complex surgical environments.

## Supplementary Information

Below is the link to the electronic supplementary material.

Supplementary file 1 (pdf 1365 KB)

## Data Availability

We plan to release our code at https://github.com/giiinger98/DyEndoVO for reproducibility.

## References

[1] Campos C, Elvira R, Rodríguez JJG, Montiel JM, Tardós JD (2021) ORB-SLAM3: An accurate open-source library for visual, visual-inertial, and multimap SLAM. IEEE Trans Rob 37(6):1874–1890

[2] Whelan T, Leutenegger S, Salas-Moreno RF, Glocker B, Davison AJ (2015) ElasticFusion: Dense SLAM without a pose graph. In: Robotics: Science and Systems (RSS), vol 11. Rome

[3] Xie J, Xie W, Zisserman A (2022) Segmenting moving objects via an object-centric layered representation. Adv Neural Inform Process Sys 35:28023–28036

[4] Hayoz M, Hahne C, Gallardo M, Candinas D, Kurmann T, Allan M, Sznitman R (2023) Learning how to robustly estimate camera pose in endoscopic videos. Int J Computer Assist Radiol Surg, 1–8 10.1007/s11548-023-02919-w PMC10329609 37184768

[5] Kendall A, Grimes M, Cipolla R (2015) Posenet: A convolutional network for real-time 6-dof camera relocalization. In: Proc IEEE Int Conf Computer Vision (ICCV), pp 2938–2946

[6] Laskar Z, Melekhov I, Kalia S, Kannala J (2017) Camera relocalization by computing pairwise relative poses using convolutional neural network. In: Proceedings of the IEEE Int Conf Computer Vision (ICCV) Workshops, pp 929–938

[7] Ding M, Wang Z, Sun J, Shi J, Luo P (2019) CamNet: Coarse-to-fine retrieval for camera re-localization. In: Proc IEEE/CVF Int Conf Computer Vision (ICCV), pp 2871–2880

[8] Engel J, Schöps T, Cremers D (2014) LSD-SLAM: Large-scale direct monocular SLAM. In: European Conference on Computer Vision (ECCV), pp. 834–849. Springer

[9] Parameshwara CM, Hari G, Fermüller C, Sanket NJ, Aloimonos Y (2022) Diffposenet: Direct differentiable camera pose estimation. In: Proc IEEE/CVF Conf Computer Vision Pattern Recogn (CVPR), pp 6845–6854

[10] Campbell D, Liu L, Gould S (2020) Solving the blind perspective-n-point problem end-to-end with robust differentiable geometric optimization. In: Computer Vision–ECCV 2020: 16th European Conference, Glasgow, UK, August 23–28, 2020, Proceedings, Part II 16, pp. 244–261. Springer

[11] Gould S, Hartley R, Campbell D (2021) Deep declarative networks. IEEE Trans Pattern Anal Mach Intell 44(8):3988–4004 10.1109/TPAMI.2021.3059462 33591908

[12] Brachmann E, Krull A, Nowozin S, Shotton J, Michel F, Gumhold S, Rother C (2017) DSAC: Differentiable RANSAC for camera localization. In: Proc IEEE Conf Computer Vision Pattern Recogn (CVPR), pp 6684–6692

[13] Teed Z, Deng J (2021) Droid-SLAM: Deep visual SLAM for monocular, stereo, and RGB-D cameras. Adv Neural Inform Process Sys 34:16558–16569

[14] Teed Z, Lipson L, Deng J (2023) Deep patch visual odometry. Adv Neural Inform Process Syst 36:39033–39051

[15] Zhong F, Wang S, Zhang Z, Wang Y (2018) Detect-SLAM: Making object detection and SLAM mutually beneficial. In: 2018 IEEE Winter Conf Appl Computer Vision (WACV), pp 1001–1010. IEEE

[16] Bescos B, Fácil JM, Civera J, Neira J (2018) DynaSLAM: Tracking, mapping, and inpainting in dynamic scenes. IEEE Robot Autom Lett 3(4):4076–483

[17] Yu C, Liu Z, Liu X-J, Xie F, Yang Y, Wei Q, Fei Q (2018) DS-SLAM: A semantic visual SLAM towards dynamic environments. In: 2018 IEEE/RSJ Int Conf Intell Robots Syst (IROS), pp 1168–1174. IEEE

[18] Zhang T, Zhang H, Li Y, Nakamura Y, Zhang L (2020) Flowfusion: Dynamic dense RGB-D SLAM based on optical flow. In: 2020 IEEE Int Conf Robot Autom (ICRA), pp 7322–7328. IEEE

[19] Marmol A, Banach A, Peynot T (2019) Dense-ArthroSLAM: Dense intra-articular 3-d reconstruction with robust localization prior for arthroscopy. IEEE Robot Autom Lett 4(2):918–925

[20] Grasa OG, Bernal E, Casado S, Gil I, Montiel J (2013) Visual SLAM for handheld monocular endoscope. IEEE Trans Med Imag 33(1):135–146 10.1109/TMI.2013.2282997 24107925

[21] Mahmoud N, Collins T, Hostettler A, Soler L, Doignon C, Montiel JMM (2018) Live tracking and dense reconstruction for handheld monocular endoscopy. IEEE Trans Med Imag 38(1):79–89 10.1109/TMI.2018.2856109 30010552

[22] Song J, Zhang R, Zhu Q, Lin J, Ghaffari M (2024) BDIS-SLAM: A lightweight CPU-based dense stereo SLAM for surgery. Int J Comput Assist Radiol Surg 19(5):811–820 38238493 10.1007/s11548-023-03055-1

[23] Lamarca J, Parashar S, Bartoli A, Montiel J (2020) Defslam: Tracking and mapping of deforming scenes from monocular sequences. IEEE Trans Rob 37(1):291-303

[24] Song J, Wang J, Zhao L, Huang S, Dissanayake G (2018) MIS-SLAM: Real-time large-scale dense deformable SLAM system in minimal invasive surgery based on heterogeneous computing. IEEE Robot Autom Lett 3(4):4068–4075

[25] Sengupta A, Bartoli A (2021) Colonoscopic 3D reconstruction by tubular non-rigid structure-from-motion. Int J Comput Assist Radiol Surg 16(7):1237–1241 34031817 10.1007/s11548-021-02409-x

[26] Zhou H, Jayender J (2021) Emdq-slam: Real-time high-resolution reconstruction of soft tissue surface from stereo laparoscopy videos. In: Medical Image Computing and Computer Assisted Intervention–MICCAI 2021: 24th International Conference, Strasbourg, France, September 27–October 1, 2021, Proceedings, Part IV 24, pp 331–340. Springer 10.1007/978-3-030-87202-1_32 PMC9165607 35664445

[27] Rodriguez JJG, Montiel JM, Tardós JD (2024) NR-SLAM: Nonrigid monocular SLAM. IEEE Trans Rob 40:4252–4264

[28] Li Q, Yang S, Shen D, Jin Y (2024) Free-DyGS: Camera-Pose-Free Scene Reconstruction based on Gaussian Splatting for Dynamic Surgical Videos. arXiv preprint

[29] Wang K, Yang C, Wang Y, Li S, Wang Y, Dou Q, Yang X, Shen W (2024) Endogslam: Real-time dense reconstruction and tracking in endoscopic surgeries using gaussian splatting. In: Int Conf Med Image Comput Computer-Assis Intervent (MICCAI), pp 219–229. Springer

[30] Kerbl B, Kopanas G, Leimkühler T, Drettakis G (2023) 3D gaussian splatting for real-time radiance field rendering. ACM Trans Graph 42(4):139–1

[31] Teed Z, Deng J (2020) RAFT: Recurrent all-pairs field transforms for optical flow. In: Computer Vision–ECCV 2020: 16th European Conference, Glasgow, UK, August 23–28, 2020, Proceedings, Part II 16, pp. 402–419. Springer

[32] Shvets AA, Rakhlin A, Kalinin AA, Iglovikov VI (2018) Automatic instrument segmentation in robot-assisted surgery using deep learning. In: 2018 17th IEEE Int Conf Mach Learn Appl (ICMLA), pp 624–628. IEEE

[33] Hong W-Y, Kao C-L, Kuo Y-H, Wang J-R, Chang W-L, Shih C-S (2020) Cholecseg8k: a semantic segmentation dataset for laparoscopic cholecystectomy based on cholec80. arXiv preprint arXiv:2012.12453

[34] Allan M, Mcleod J, Wang C, Rosenthal JC, Hu Z, Gard N, Eisert P, Fu KX, Zeffiro T, Xia W, et al (2021) Stereo correspondence and reconstruction of endoscopic data challenge. arXiv preprint arXiv:2101.01133

[35] Recasens D, Lamarca J, Fácil JM, Montiel JM, Civera J (2021) Endo-Depth-and-Motion: Reconstruction and tracking in endoscopic videos using depth networks and photometric constraints. IEEE Robo Autom Lett 6(4):7225–7232

