# Supplementary material for: DyEndoVO: scene dynamics-aware pose estimation of endoscope in minimally invasive surgery
Source: Int J Comput Assist Radiol Surg. 2025 Dec 22;21(4):723–33. doi: 10.1007/s11548-025-03549-0 (PMC13194247; doi:10.1007/s11548-025-03549-0)
Supplement: Supplementary file 1 — (pdf 1365 KB) [file 11548_2025_3549_MOESM1_ESM.pdf]

DyEndoVO: Scene Dynamics-Aware Pose Estimation of Endoscope in Minimally Invasive Surgery

Jinjing Xu[1,4*], Reuben Docea[1,2,4], Micha Pfeiffer[1,4], Martin Wagner[3], Marius Distler[3], Stefanie Speidel[1,2,3,4]

[1*]National Center for Tumor Diseases (NCT/UCC), Dresden, Germany.
[2*]The Centre for Tactile Internet (CeTI), TU Dresden, Dresden, Germany.
[3*]University Hospital Carl Gustav Carus, Dresden, Germany.
[4*]German Cancer Research Center(DKFZ), Heidelberg, Germany.

*Corresponding author(s). E-mail(s): jinjing.xu@nct-dresden.de;
Contributing authors: reuben.docea@nct-dresden.de;
micha.pfeiffer@nct-dresden.de; martin.wagner@ukdd.de;
marius.distler@uniklinikum-dresden.de; stefanie.speidel@nct-dresden.de;

A Details in Generation of DynaSCARED

A.1 Example data across scenarios

Please refer to DynaSCARED dataset for samples in our dataset. We highly recommend checking out the contained video for viewing the modeled scene dynamics, especially for the tissue deformation. Below we clarify details in critical components of our data generation pipeline.

A.2 Modelling rigid movement of surgical tools

The surgical tools patches are extracted in sampled frame from instrument segmentation dataset Robotic Instrument Segmentation [1] (RIS), and the rigid movement are simulated by performing homography transformations on the extracted image plane. In details, we first sample a source image frame and extract the contained tool patch based on ground truth tool masks; randomness is again enforced in the extraction by

the sampling of tool queries. The extracted patch P_{tool} is taken as the starting status of the foreground tool. We then constructed N homography matrices composed of random translation, rotations, scaling and perspective distortions, each homography are accumulated with previous ones before being applied on the image plane P_{tool} for realistic and smooth tool movement.

A.3 Modelling non-rigid tissue deformation

The soft tissue patches are retrieved from semantic segmentation dataset CholecSeg8k [2]. As the strategy adopted above, we extracted the initial tissue patch from sampled image with queried label randomly chosen from 8 classes of tissue structure. Follwoing the strategy in [3], we leverage thin plate splines to simulate the non-rigidity in soft tissue deformation. Thin plate splines define a non-linear coordinate transformation with a set of control points, we adopt the uniform grid (grid size as 40) distributed over the tissue patch as the control points, and perturbed them with randomly sampled distances $d_i \in (0, 5)$, where D is the grid size; then model the non-linear transformations based on the control points and applied on the tissue patch to mimic tissue deformation.

A.4 Camera motion and formulation of stereo pairs

To imitate the real motion trajectory of handheld endoscope, we obtain the background frames and the corresponding camera poses from stereo reconstruction dataset SCARED[4]. The captured scenes in this dataset are visually rigid, therefore we can formulate ground truth motion masks purely based on the status of simulated surgical tool and soft tissues. Stereo pairs of background images can be easily extracted from the source dataset, nevertheless, the overlaid foreground objects in the stereo pairs are only correspondent up to an disparity map. To tackle this problem, we assume the moved foreground 2D patch plane is always facing the front of the camera throughout the sequence, and we independently sample random depth value D_i for each foreground moved object P_i, the disparity for the object can be obtained based on depth D_i, given camera known intrinsic and extrinsic (stereo baseline).

B Visualisation of Inferenced Weight Map on StereoMIS

Please find the video demo of our estimated motion maps with animated visualization in comparison with other baselines. The motion mask is overlaid on raw images for enhanced inspection.

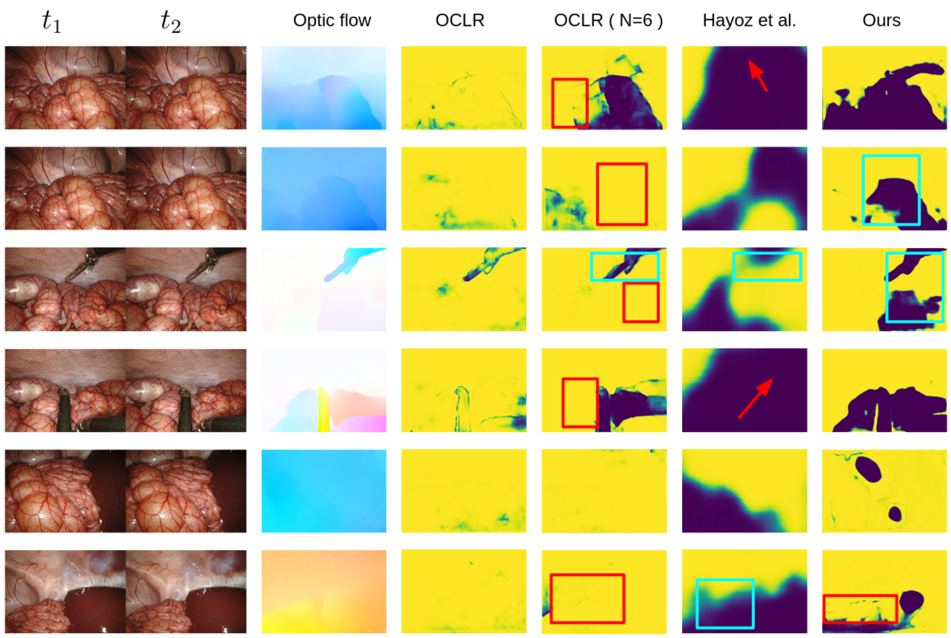

Fig. 1: **Visualization of learned weight map on StereoMIS.** We follow the same notation as in Fig. 4.

C Data Processing Approach and Supportive Evaluation Results on StereoMIS

C.1 Extraction of short snippets in StereoMIS

StereoMIS[5] is an in-vivo robotic-assisted (da Vinci Surgical System) MIS dataset labeled with ground truth endoscope poses. Our experiments are conducted on the publicly available porcine part; specifically, they are sequence P1_0 for training, sequence P2_0-P2_8 for evaluation, following the train/validation split as in [5]. Notice sequence P2_3 is excluded from the source to extract the snippets, as it contains heavy respiration, and the whole scene being captured is "breathing", leading to no valid rigid anchoring for visual odometry methods.

Notice the sequences in StereoMIS contain scene dynamics, whereas as a robotic-assisted MIS dataset, the camera are mostly still. To sufficiently benchmark VO methods, we extract short snippets captured only when the endoscope is moving. Specifically, we decide cameras motion status by comparing the relative poses between successive frames with a threshold ϕ; we set ϕ to be small enough to obtain as much moving-camera snippets, meanwhile avoiding it being tiny and wrongly extracting still-camera snippets (the ground truth trajectory contains noise, as we observe and being claimed[5]). To get short sequences rather than individual pairs of frames, we compose a snippet if there are continuously H successive frames labeled with the

same camera status after thresholding. This way we obtained 345 sequence snippets with 26 frames each ($H = 26$), and further grouped them to be liver scene (total 5174 frames) and bowel scene (total 3796 frames) as they perform different deformation patterns. Following the same approach, we obtain still-camera snippets, and group these snippets additionally based on the presence of surgical tools (without surgical tools/tool-tissue interaction) for finer analysis; specifically, we obtain 2500/1326, 1066/1248 frames for each scenario.

C.2 Evaluation on Still Camera snippets and full sequences

D Run-time Analysis

Below, we report the run-time analysis of our method. The evaluation is conducted on the in-vivo dataset StereoMIS (we use the bowel scene snippets containing camera

Table 2: **Experimental results on full sequence of StereoMIS.** We report additional performance when set the limitation for optimization iterations as 20 (by default we use 30), denoted as by *, our method constantly outperforms.

	P2_1: 4232			P2_2: 4277			P2_3: 4222			P2_4: 4280		
	RPE $[mm]\downarrow$	RPE $[deg]\downarrow$	ATE $[mm]\downarrow$	RPE $[mm]\downarrow$	RPE $[deg]\downarrow$	ATE $[mm]\downarrow$	RPE $[mm]\downarrow$	RPE $[deg]\downarrow$	ATE $[mm]\downarrow$	RPE $[mm]\downarrow$	RPE $[deg]\downarrow$	ATE $[mm]\downarrow$
Hayoz et al.[5]	0.352	0.135	107.347	0.333	0.161	44.705	1.502	0.811	39.516	0.329	0.167	51.399
Ours	0.301	0.086	18.097	0.185	0.055	46.343	0.152	0.032	16.286	0.311	0.138	43.615
Ours(rf.W)	0.298	0.088	16.020	0.183	0.053	28.803	0.149	0.030	13.699	0.299	0.133	29.922
Hayoz et al.*[5]	0.350	0.135	106.732	0.304	0.152	33.393	1.524	0.813	40.247	0.329	0.167	54.536
OCLR (N=6)*[3]	0.304	0.087	18.159	0.188	0.058	27.798	0.158	0.035	16.205	0.314	0.139	38.841
EDaM[6]	0.322	0.127	40.816	0.268	0.102	115.119	0.265	0.090	34.544	0.337	0.165	34.985
Ours*	0.301	0.086	17.935	0.185	0.055	47.051	0.152	0.032	16.275	0.311	0.138	42.789
ORBSLAM3[7]	0.493	0.273	13.134	0.417	0.203	54.244	0.352	0.124	5.450	0.522	0.313	28.642
ElasticFusion[8]	0.628	0.435	138.983	0.389	0.175	41.721	0.990	0.436	8.148	0.523	0.343	69.024
DroidSLAM[9]	0.502	0.240	31.862	0.655	0.293	5.800	0.672	0.228	15.437	0.486	0.267	8.521

	P2_5: 5367			P2_6: 2137			P2_7: 7572			P2_8: 6250		
	RPE $[mm]\downarrow$	RPE $[deg]\downarrow$	ATE $[mm]\downarrow$	RPE $[mm]\downarrow$	RPE $[deg]\downarrow$	ATE $[mm]\downarrow$	RPE $[mm]\downarrow$	RPE $[deg]\downarrow$	ATE $[mm]\downarrow$	RPE $[mm]\downarrow$	RPE $[deg]\downarrow$	ATE $[mm]\downarrow$
Hayoz et al.[5]	0.292	0.142	57.552	0.154	0.076	55.024	0.086	0.049	21.434	0.081	0.050	15.019
Ours	0.293	0.148	65.051	0.166	0.079	47.643	0.082	0.045	35.178	0.079	0.047	31.565
Ours(rf.W)	0.266	0.130	42.277	0.156	0.077	43.425	0.081	0.046	27.494	0.077	0.045	20.169
Hayoz et al.*[5]	0.291	0.141	60.202	0.154	0.076	55.320	0.086	0.049	21.333	0.081	0.050	14.919
OCLR (N=6)*[3]	0.300	0.154	57.645	0.167	0.079	45.795	0.081	0.046	33.105	0.078	0.047	33.681
EDaM[6]	0.384	0.243	61.672	0.169	0.104	23.118	0.084	0.050	36.306	0.097	0.058	28.659
Ours*	0.293	0.148	67.783	0.167	0.079	47.703	0.082	0.045	35.128	0.078	0.047	28.902
ORBSLAM3[7]	0.500	0.337	24.102	0.323	0.227	19.816	0.337	0.192	12.828	0.302	0.184	9.443
ElasticFusion[8]	0.580	0.507	91.736	0.443	0.463	54.894	0.373	0.459	59.368	0.309	0.242	23.442
DroidSLAM[9]	0.603	0.404	10.270	0.234	0.155	14.920	0.126	0.070	14.887	0.126	0.083	5.285

motion, including 3796 frames). All the experiments are conducted on an NVIDIA RTXA5000 GPU. Our method shows comparable computational time to the major baseline of Hayoz et al.[5]. However, compared to SLAM baselines, our pipeline does not achieve real-time performance, as our primary focus was on improving accuracy and robustness to dynamics, rather than on the extensive computational optimizations (like parallel multi-threading) that these SLAM systems employ.

Table 3: **Run time analysis on StereoMIS snippets.**

		Bowel: 3796 frames			
		RPE [mm]↓	RPE [deg]↓	ATE [mm]↓	FPS [Hz]↑
Visual Odometry	Hayoz et al.[5]	0.164	0.116	1.581	1.64
	EDaM[6]	**0.152**	**0.104**	1.405	1.78
	Ours	<u>0.153</u>	<u>0.106</u>	1.452	1.35
	Ours(rf.W)	**0.152**	**0.104**	1.444	1.31
SLAM	ElasticFusion[8]	0.253	0.162	1.577	28.70
	DroidSLAM[9]	0.205	0.144	<u>1.185</u>	20.82

E Failure Analysis and Limitation

While our method demonstrates a significant improvement in robustness to dynamics, we acknowledge several avenues for future work. The system's primary dependency is on optical flow quality, which is a known bottleneck in challenging MIS scenes with smoke or blur. A valuable future direction is to reduce this sensitivity, perhaps by training the network to predict its own uncertainty to down-weight unreliable optical flow. Our framework is also designed as a visual odometry (VO) pipeline and therefore does not currently implement the re-localization or loop-closure modules of a full SLAM system, which makes it susceptible to long-term drift. Similarly, it relies on a partially static ”anchor” and is not designed for fully dynamic scenes.

References

[1] Shvets, A.A., Rakhlin, A., Kalinin, A.A., Iglovikov, V.I.: Automatic instrument segmentation in robot-assisted surgery using deep learning. In: 2018 17th IEEE International Conference on Machine Learning and Applications (ICMLA), pp. 624–628 (2018). IEEE

[2] Hong, W.-Y., Kao, C.-L., Kuo, Y.-H., Wang, J.-R., Chang, W.-L., Shih, C.-S.: Cholecseg8k: a semantic segmentation dataset for laparoscopic cholecystectomy based on cholec80. arXiv preprint arXiv:2012.12453 (2020)

[3] Xie, J., Xie, W., Zisserman, A.: Segmenting moving objects via an object-centric layered representation. In: Advances in Neural Information Processing Systems, vol. 35, pp. 28023–28036 (2022)

[4] Allan, M., Mcleod, J., Wang, C., Rosenthal, J.C., Hu, Z., Gard, N., Eisert, P., Fu, K.X., Zeffiro, T., Xia, W., et al.: Stereo correspondence and reconstruction of endoscopic data challenge. arXiv preprint arXiv:2101.01133 (2021)

[5] Hayoz, M., Hahne, C., Gallardo, M., Candinas, D., Kurmann, T., Allan, M., Sznitman, R.: Learning how to robustly estimate camera pose in endoscopic videos. International Journal of Computer Assisted Radiology and Surgery, 1–8 (2023)

[6] Recasens, D., Lamarca, J., Fácil, J.M., Montiel, J.M., Civera, J.: Endo-Depth-and-Motion: Reconstruction and tracking in endoscopic videos using depth networks and photometric constraints. IEEE Robotics and Automation Letters **6**(4), 7225–7232 (2021)

[7] Campos, C., Elvira, R., Rodríguez, J.J.G., Montiel, J.M., Tardós, J.D.: ORB-SLAM3: An accurate open-source library for visual, visual–inertial, and multimap SLAM. IEEE Transactions on Robotics **37**(6), 1874–1890 (2021)

[8] Whelan, T., Leutenegger, S., Salas-Moreno, R.F., Glocker, B., Davison, A.J.: ElasticFusion: Dense SLAM without a pose graph. In: Robotics: Science and Systems (RSS), vol. 11 (2015). Rome

[9] Teed, Z., Deng, J.: Droid-SLAM: Deep visual SLAM for monocular, stereo, and RGB-D cameras. In: Advances in Neural Information Processing Systems, vol. 34, pp. 16558–16569 (2021)
